# Time Course of Acute Vertebral Fractures: A Prospective Multicenter Cohort Study

**DOI:** 10.3390/jcm10245961

**Published:** 2021-12-19

**Authors:** Hiroyuki Inose, Tsuyoshi Kato, Shinichi Shirasawa, Shinji Takahashi, Masatoshi Hoshino, Yu Yamato, Yu Matsukura, Takashi Hirai, Toshitaka Yoshii, Atsushi Okawa

**Affiliations:** 1 Department of Orthopedic and Trauma Research, Graduate School, Tokyo Medical and Dental University, Tokyo 108-0075, Japan; 2 Department of Orthopaedics, Ome Municipal General Hospital, Tokyo 198-0042, Japan; katoorth@gmail.com; 3 Department of Orthopaedics, Suwa Central Hospital, Nagano 391-8503, Japan; sshirasawa@aol.com; 4 Department of Orthopedic Surgery, Graduate School of Medicine, Osaka City University, Osaka 545-8585, Japan; shinji@med.osaka-cu.ac.jp; 5 Department of Orthopedic Surgery, Osaka City General Hospital, Osaka 534-0021, Japan; hoshino717@gmail.com; 6 Division of Geriatric Musculoskeletal Health, Hamamatsu University School of Medicine, Shizuoka 431-3192, Japan; yamato@hama-med.ac.jp; 7 Department of Orthopaedics, Graduate School, Tokyo Medical and Dental University, Tokyo 108-0075, Japan; matsukura.orth@tmd.ac.jp (Y.M.); hirai.orth@tmd.ac.jp (T.H.); yoshii.orth@tmd.ac.jp (T.Y.); okawa.orth@tmd.ac.jp (A.O.)

**Keywords:** osteoporotic vertebral fracture, residual pain, visual analog scale, quality of life

## Abstract

To date, it is still unclear how fresh osteoporotic vertebral fractures (OVFs) affect the patient’s quality of life and low back pain during a follow-up period of more than 1 year. In the previous trial, women with fresh OVF were randomized to rigid or soft brace for 12 weeks, then both groups were followed for the subsequent 48 weeks. In women completing this trial at our affiliated hospitals, we conducted a follow-up study to investigate the long-term course of an acute vertebral fracture in terms of pain and quality of life. When comparing visual analog scale scores for low back pain and European Quality of Life-5 Dimensions Questionnaire scores between consecutive time points, a significant difference was found between 0 and 12 weeks, but not between 12 and 48 weeks or between 48 weeks and final follow-up. A total 25% had residual low back pain at the final follow-up. A stepwise logistic regression analysis identified age and previous vertebral fracture as predictors of residual low back pain at the final follow-up. Therefore, the degree of low back pain and impairment of the quality of life improved by 12 weeks after injury and did not change thereafter until a mean follow-up of 5.3 years.

## 1. Introduction

Vertebral fractures are the most common osteoporotic fracture [1]. When osteoporotic vertebral body fractures occur, the symptoms improve approximately 3 months after the injury in most cases [2]. However, in some cases, the symptoms persist chronically. A study found that patients with new vertebral fractures had significantly more back pain and poorer physical function at all time points up to 12 months after fracture than those without fractures [2]. In addition, if there is a history of vertebral fractures, recovery after a new vertebral fracture is even worse. In a study comparing the post-vertebral fracture course, patients with a history of vertebral fracture had significantly lower physical motor function, activities of daily living, and quality of life (QOL) up to 12 months after injury than patients without a history of vertebral fracture [3]. However, it is still unclear how fresh vertebral fractures affect the patient’s QOL and low back pain during a follow-up period of more than 1 year. Thus, this study aimed to describe the course of acute vertebral fractures in terms of pain and QOL and to characterize patients with residual low back pain long after a vertebral fracture.

## 2. Materials and Methods

This study was a follow-up study of women involved in the previous prospective randomized study (UMIN000014876) that compared the effectiveness of rigid and soft braces for acute thoracolumbar OVFs [4]. Briefly, the original trial enrolled 284 patients aged between 65 and 85 years who were diagnosed with one fresh OVF between T10 and L2 within four weeks of injury; 141 of these patients were randomly assigned to wear rigid braces and 143 were assigned to wear soft braces. Patients wore ready-made braces until a custom-made thoracolumbar sacral rigid or soft brace was applied. Patients in the rigid-brace group received a rigid thoracolumbosacral orthosis. Patients in the soft-brace group received a soft thoracolumbosacral orthosis. In both the rigid and flexible bracing groups, the patients were instructed to always wear the braces, when possible. All the participants were instructed to wear the brace for a total of 12 weeks. Detailed inclusion and exclusion criteria of the study have been described previously [4].

Among the patients who completed the previous brace trial, patients from hospitals that agreed to participate in this study were included in this study. Accordingly, a total of 73 patients were enrolled. Of the 73, 3 died, 2 refused to cooperate, and 28 could not be contacted. Finally, 40 patients with mean 5.3 years of follow-up were included in this study. With regard to the use of anti-osteoporosis treatments during the 48-week brace treatment prospective randomized study, the patients were allowed to use only the medications that were used prior to the injury or newly prescribed active vitamin D [4]. During the subsequent follow-up period, prescription of any anti-osteoporosis medication was allowed.

This study was approved by each hospital’s institutional review board, and informed consent was obtained from all the participants included in the study.

### 2.1. Patient-Reported Outcome Measures

Regarding the patient-reported outcome measures (PROMs), scores on the European Quality of Life-5 Dimensions (EQ-5D; range, −0.111 to 1, with higher scores indicating a better QOL) [5] and the visual analog scale (VAS) for low back pain (range, 0–10, with higher scores indicating more severe pain) [6] were used. These questionnaires were provided at a regular hospital visit (0, 12, and 48 weeks after brace application) but were completed without assistance from the surgeon or any other person involved in this study. After 48 weeks, since regular visits to the hospital were not mandatory, outcome assessment at the last follow-up was completed by mailing a questionnaire. To maximize participant retention, we decided to mail the questionnaires. This is because, according to previous research, comparing three different methods of administering a brief screening questionnaire to the elderly, response rates were higher for the postal questionnaire than the interview method [7].

### 2.2. Radiographic Assessment

Lateral radiography was performed at 0, 12, and 48 weeks. MRI was performed at enrollment. In the radiographic analysis, the anterior vertebral body compression percentage [4,8], which is defined as the ratio between the vertical height of the compressed anterior section of the injured vertebral body and the posterior vertebral body height at the same level, was measured independently at 0, 12, and 48 weeks after brace application by two radiologists. The mean values of the two evaluators were used. In this study, a previous vertebral fracture was defined as a decrease of at least 20% in the height of any vertebral body at Week 0 [9]. To investigate the presence of degenerative spinal diseases that can cause low back pain, we investigated lumbar spinal canal stenosis and lumbar disc herniation by MRI at enrollment. Lumbar spinal canal stenosis was diagnosed as C or higher in Schizas’ classification [10].

### 2.3. Data Analysis

All data were collected by a clinical research assistant. An analysis of variance with repeated measures was used to analyze the data over time. When there was a significant main effect of time, Tukey’s HSD analysis was performed to identify the differences among time points.

In this study, “residual low back pain” was defined as VAS for low back pain ≥3.5 at the final follow-up; VAS score <3.5 is used to describe mild pain, and VAS score ≥ 3.5 is used to describe moderate or severe pain [11]. We performed outcome and risk factor analyses by comparing patients with VAS scores <3.5 and ≥3.5 for low back pain. We analyzed the differences between the two groups using the Mann–Whitney U test for continuous variables and Fisher’s exact test or chi-squared test for nominal variables. To identify the most significant risk factors for residual low back pain at the final follow-up, we performed risk factor analysis using multivariable logistic regression analysis with a forward-backward stepwise procedure (*p* < 0.1 for entry). We then calculated the odds ratios (ORs) and their approximate 95% confidence intervals (CIs) for residual low back pain. For continuous variables, the OR reflects the incremental risk associated with a one-unit change in that variable. JMP version 12 (SAS Institute, Cary, NC, USA) was used for all statistical analyses. All tests were two-sided, and *p*-values < 0.05 were considered significant.

## 3. Results

### 3.1. Demographics

**Figure 1:**
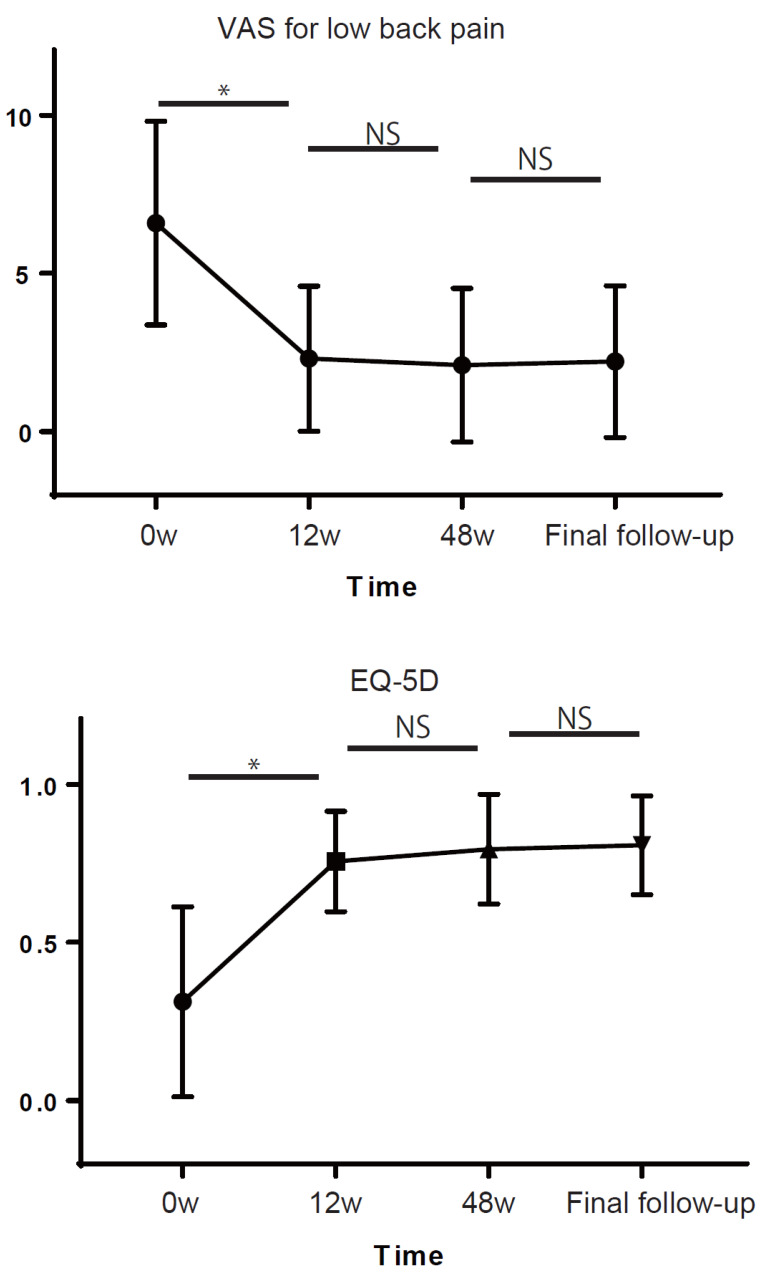
Temporal trends in outcome measures. The visual analog scale (VAS) for low back pain (0–10, with higher scores indicating severe pain) and the European Quality of Life-5 Dimensions Questionnaire (EQ-5D, −0.111 to 1, with higher scores indicating better quality of life). Means with standard deviations at baseline and each follow-up are shown. * *p* < 0.05, NS not significant.

A total of 40 patients with a mean follow-up of 5.3 years were included in this study. The mean age was 73.9 years. Figure 1 shows the time course of VAS for low back pain and EQ-5D after OVF. Time had a significant main effect on VAS for low back pain and EQ-5D (*p* < 0.001 and *p* < 0.001, respectively). Comparison of VAS scores for low back pain between consecutive time points showed a significant difference between 0 and 12 weeks (*p* < 0.001), but not between 12 and 48 weeks (*p* = 0.97), or between 48 weeks and final follow-up (*p* = 0.99) (Figure 1). Comparison of EQ-5D scores between consecutive time points showed a significant difference between 0 and 12 weeks (*p* < 0.001), but not between 12 and 48 weeks (*p* = 0.82), or between 48 weeks and final follow-up (*p* = 0.99) (Figure 1).

### 3.2. Characteristics of Patients with Residual Low Back Pain at 5 Years after OVF

We then divided the patients into two groups according to their VAS score at the last follow-up: the residual low back pain group and the no low back pain group. Of the 40 patients analyzed in this study, 10 (25.0%) reported residual low back pain at a mean 5.3 years after OVFs. The baseline characteristics of the patients are shown in Table 1. In the residual low back pain group, the patients were older, and the percentage of patients with a history of pre-existing vertebral fracture was higher. No significant differences were observed in the other background variables between the two groups.

Table 2 shows the differences in PROMs between the groups with VAS scores < 3.5 and ≥3.5 at the final follow-up. VAS scores for low back pain did differ not significantly between the two groups at 0 and 12 weeks, but were significantly worse in the residual low back pain group at 48 weeks and final follow-up (*p* < 0.001 and <0.001, respectively). The EQ-5D score was not significantly different between the two groups at 0 and 12 weeks, but was significantly worse in the residual low back pain group at 48 weeks and final follow-up (*p* < 0.001 and *p* = 0.001, respectively). We then examined the trends in the VAS score for low back pain in the residual low back pain group and no low back pain group. In the no low back pain group, a significant difference was found between VAS scores at 0 and 12 weeks (*p* < 0.001), but no significant difference was noted in the VAS scores between 12 and 48 weeks or between 12 weeks and final follow-up (*p* = 0.41 and 0.30, respectively). In the residual low back pain group, no significant difference was found in the VAS scores between 12 and 48 weeks (*p* = 0.08), but a significant difference was noted in the VAS scores between 0 and 12 weeks and between 12 weeks and final follow-up (*p* < 0.001 and *p* = 0.02, respectively).

Table 3 shows the differences in the radiographic assessment between the groups with VAS scores of <3.5 and ≥3.5. No significant difference was observed in the anterior vertebral body compression percentage between the two groups, although there was a trend toward lower anterior vertebral body compression percentage in the residual low back pain group throughout the period from 0 to 48 weeks (*p* = 0.17, 0.11, and 0.09, respectively).

Lastly, the predictors at 12 weeks after OVFs for residual low back pain at the final follow-up were evaluated using a stepwise multiple logistic regression analysis (Table 4). Based on the univariate analysis, the dependent variable was defined as the presence of residual low back pain at the final follow-up, and the independent variables were age, previous vertebral fracture, and EQ-5D score at 12 weeks after OVF. As a result, the independent risk factors at 12 weeks were identified as age (OR = 1.19; 95% CI, 1.01–1.46; *p* = 0.04) and previous vertebral fracture (OR = 6.28; 95% CI, 1.24–39.83; *p* = 0.03).

## 4. Discussion

This study investigated the course of acute vertebral fracture in terms of pain and QOL. When comparing VAS for low back pain and EQ-5D scores between consecutive time points, a significant difference was observed between 0 and 12 weeks, but not between 12 and 48 weeks or between 48 weeks and final follow-up. Twenty-five percent of patients had residual low back pain at the final follow-up. The patients with residual low back pain after OVF had a higher percentage of pre-existing vertebral fractures and were older than those who did not have residual low back pain. A stepwise logistic regression analysis identified age and previous vertebral fracture as predictors of residual low back pain at the final follow-up.

This study showed that when comparing VAS scores for low back pain and EQ-5D scores between consecutive time points, a significant difference was found between 0 and 12 weeks, but not between 12 and 48 weeks or between 48 weeks and final follow-up. This result is consistent with previous reports that pain improved by 3 months after the fracture and did not change significantly until 12 months thereafter [12]. Collectively, these results suggest that if severe pain remains after the acute phase, it might be unlikely that the pain will improve spontaneously.

Among patients with acute vertebral fractures, 25% had mild or severe low back pain for an average of 5.3 years after injury based on VAS for low back pain. Patients with mild or severe low back pain were older and had a higher percentage of patients with pre-existing vertebral fractures than those with moderate or no pain. The results were partially consistent with a previous report stating that chronic pain after acute spine fractures was only maintained in patients with multiple compression fractures, reduced height, and low bone density [13].

In this study, the VAS score for low back pain was not significantly different between the residual low back pain group and no low back pain group at 0 and 12 weeks, but was significantly worse in the residual low back pain group at 48 weeks and final follow-up. Regarding the transition of pain within the group, although not significant, the low back pain tended to improve after 12 weeks in the no low back pain group. By contrast, back pain deteriorated after 12 weeks in the residual low back pain group. In a randomized controlled trial comparing vertebroplasty and conservative treatment for patients with vertebral fractures who reported severe pain for more than 3 months, vertebroplasty was associated with better pain relief and improved functional outcomes at 1 year compared with conservative treatment [14]. Therefore, taking into account the improvement of pain in patients who report severe low back pain 3 months after a vertebral fracture, vertebroplasty should be considered rather than conservative treatment. This treatment strategy should be tested in the future.

Since OVF-induced pain significantly improves by 12 weeks, we decided to investigate predictors for residual low back pain at 12 weeks after OVF. A stepwise logistic regression analysis identified age and previous vertebral fracture as predictors for residual low back pain at a mean of 5.3 years after OVFs. Therefore, when a new vertebral fracture occurs in an older patient with a pre-existing vertebral fracture, the patient is likely to have residual low back pain in the future. Furthermore, risk factors for OVFs include older age, low bone mineral density, and pre-existing vertebral fractures [15]. Therefore, elderly patients with new OVF and pre-existing vertebral fractures are at risk of further subsequent fractures. In this study, we do not know whether subsequent vertebral fractures occurred in this group of patients after 48 weeks, because imaging evaluation was not performed in the final follow-up. According to a post-hoc analysis of the original prospective study, patients with subsequent vertebral fractures at Week 48 had significantly more severe low back pain than those without subsequent fractures at Week 48 [16]. Therefore, in this study, it cannot be ruled out that the presence of subsequent vertebral fractures at the time of the final follow-up may be associated with residual low back pain. However, if a new OVF occurs in an older patient with a pre-existing vertebral fracture, it may be desirable to provide intensive osteoporosis treatment to prevent subsequent fractures. Further research is needed to determine which osteoporosis drugs are the most effective in reducing subsequent fractures in elderly patients with pre-existing vertebral fractures.

This study had some limitations. First, several patients were excluded after enrollment which might have led to a slight decrease in the sample size. Accordingly, attrition bias may limit the internal validity of this study. Second, we did not investigate the bone mineral density in this study. Although it is undeniable that the severity of osteoporosis may affect back pain, a decrease in bone mineral density does not necessarily lead to an increase in low back pain. In fact, the authors of several studies concluded that there is no evidence supporting a relationship between low back pain and bone mineral density [17,18]. Third, given the small percentage of patients who continued to attend the hospital, no radiographic evaluation was performed at the last follow-up. This prevented us from assessing the relationship between residual low back pain and non-union, subsequent fractures, and spinal alignment at the final follow-up. Lastly, the results of the multiple logistic regression analysis showed that there were two independent variables. Accordingly, the event per variable (EPV) was five in this model. However, the rule of thumb of 10 or more EPV in logistic models is not a well-defined bright line [19]. A simulation study showed that statistical problems are uncommon with 5–9 EPV, and still observed with 10–16 EPV [19]. Further studies are required to address these limitations and to validate our findings.

## 5. Conclusions

This study demonstrated that the degree of pain and impairment of QOL after OVF improved by 12 weeks after injury and did not change thereafter, until a mean follow-up period of 5.3 years. In addition, patients with residual low back pain after OVF had a higher percentage of pre-existing vertebral fractures and were older than those who did not have residual low back pain.

## Figures and Tables

**Table 1:** Baseline characteristics of the patients.

Characteristics	VAS < 3.5 (*n* = 30)	VAS ≥ 3.5 (*n* = 10)	*p* Value
Age (years)	72.8 ± 5.5	77.1 ± 4.8	0.03 *
Receiving osteoporosis therapy at enrollment	5 (17)	3 (30)	0.38
Any previous vertebral fracture	6 (20)	6 (60)	0.04 *
Spinal disorders	9 (30)	1 (10)	0.40
Lumbar canal stenosis	8 (27)	0 (0)	
Lumbar disc hernia	1 (3)	1 (10)	
Level			0.70
T10	1 (3)	0 (0)	
T11	2 (7)	0 (0)	
T12	7 (23)	3 (30)	
L1	11 (37)	3 (30)	
L2	9 (30)	4 (40)	
Type of brace			
Rigid	15 (50)	4 (40)	0.58
Soft	15(50)	6 (60)	
Follow-up period, days	1922 ± 255	1898 ± 213	0.75
Receiving osteoporosis therapy at final follow-up	13 (43)	4 (40)	0.85

Data are presented as mean ± standard deviation or *n* (%). * *p* < 0.05. VAS, visual analog scale.

**Table 2:** Patient-reported outcome measures.

Characteristic	VAS < 3.5 (*n* = 30)	VAS ≥ 3.5 (*n* = 10)	*p*
EQ-5D			
Week 0	0.31 ± 0.33	0.31 ± 0.23	0.78
12 weeks	0.78 ± 0.16	0.68 ± 0.14	0.09
48 weeks	0.85 ± 0.15	0.61 ± 0.08	<0.001 *
Final follow-up	0.85 ± 0.14	0.65 ± 0.07	0.001 *
VAS low back pain			
Week 0	6.1 ± 3.4	8.0 ± 2.5	0.08
12 weeks	2.1 ± 2.4	2.7 ± 2.1	0.30
48 weeks	1.2 ± 1.6	5.1 ± 2.3	<0.001 *
Final follow-up	1.1 ± 1.3	5.7 ± 1.5	<0.001 *

* *p* < 0.05. VAS, visual analog scale; EQ-5D, European Quality of Life-5 Dimensions.

**Table 3:** Radiographic assessment.

Characteristic	VAS < 3.5 (*n* = 30)	VAS ≥ 3.5 (*n* = 10)	*p* Value
Anterior Vertebral Body Compression Percentage			
0 week	74.6 ± 12.6	65.4 ± 20.3	0.17
12 weeks	62.4 ± 15.6	51.5 ± 15.1	0.11
48 weeks	61.9 ± 16.2	50.5 ± 18.0	0.09

Data are presented as mean ± standard deviation or *n* (%). *p* < 0.05. VAS, visual analog scale.

**Table 4:** Multiple logistic regression analysis: independent risk factors of residual low back pain (VAS ≥ 3.5 at final follow-up).

Variable	Odds Ratio	95% Confidence Interval	*p*
12 weeks			
History of vertebral fracture	6.28	1.24–39.83	0.03 *
Age	1.19	1.01–1.46	0.04 *

* *p* < 0.05. VAS, visual analog scale.

## Data Availability

Not available.
